# Diet and Common Mental Disorders: The Imperative to Translate Evidence into Action

**DOI:** 10.3389/fpubh.2016.00081

**Published:** 2016-04-29

**Authors:** Sarah R. Dash, Adrienne O’Neil, Felice N. Jacka

**Affiliations:** ^1^IMPACT Strategic Research Centre, School of Medicine, Deakin University, Geelong, VIC, Australia; ^2^School of Public Health and Preventive Medicine, Monash University, Prahran, VIC, Australia; ^3^Alfred Hospital, School of Public Health and Preventive Medicine, Prahran, VIC, Australia; ^4^Centre for Adolescent Health, Murdoch Children’s Research Centre, Parkville, VIC, Australia; ^5^Department of Psychiatry, Royal Melbourne Hospital, University of Melbourne, Melbourne, VIC, Australia; ^6^Black Dog Institute, Prince of Wales Hospital, Randwick, NSW, Australia

**Keywords:** diet, mental health, public health, prevention, epidemiology

## Introduction

The globalization of the food industry has lead to substantial dietary changes across developed and developing economies, comprising a shift toward the consumption of higher energy, less nutritious foods at the expense of traditional, more healthful, dietary patterns (1). These dietary changes have led to clear public health challenges as the burden of obesity and other diet-related non-communicable disorders (NCDs) continue to rise. In 2015, the Global Burden of Disease study identified unhealthy diet as the leading cause of early mortality worldwide (2). At the same time, mental and substance use disorders are recognized as the leading contributors to global disability (3). Of these, the common mental disorders (CMDs) – depression and anxiety – contribute the greatest proportion of disability, accounting for 40.5 and 14.6% of disease burden respectively. Only recently has it been recognized that unhealthy diet and CMDs are related: unhealthy diet is a significant risk factor not only for NCDs, such as cardiovascular diseases, some cancers, and diabetes, but also for CMDs (4). Dietary interventions may, thus, provide a far-reaching and low risk public health opportunity for the prevention and treatment of CMDs.

Traditionally, psychiatric epidemiology has directed much of its research efforts into understanding the etiology of psychiatric conditions and has lagged behind in the development of public health strategies for primary prevention (5). While the past decade has given rise to public health campaigns directed at mental illness, such campaigns are often focused on raising awareness and reducing stigma rather than on specific actions (6). Moreover, while several critical windows of opportunity for mental disorder prevention have been presented (7), there currently exists no clear or specific prevention strategy or recommendations for mental illness akin to that which exists for other common NCDs. Funding resources allocated to primary prevention of CMDs are greatly disproportionate to its disease burden, and resources for mental health prevention are not equitable to the priority placed on them by major stakeholders (8, 9). This paper argues the necessity of translating the new knowledge regarding the diet–depression paradigm into the development and implementation of public health and clinical intervention strategies at a population level.

## CMDs and Diet

There is now consistent epidemiological evidence for associations between measures of habitual diet quality and depression, globally (10–14) and across the lifespan (15–18), which do not appear to be explained by socioeconomic circumstances (4) or reverse causality (11, 19, 20). In fact, dietary habits have now been identified as a modifiable risk factor for depression and anxiety in several recent systematic reviews (21–23). The literature suggests that a good quality diet is characterized by high consumption of fruits, vegetables, whole grains, nuts, seeds, and fish while limiting intake of processed foods (24). Evidence at the clinical trial level indeed provide promising data; two new interventions indicate that preventing depression using dietary improvement as a strategy is possible (25, 26). Another systematic review has identified preliminary evidence for dietary improvement as a treatment strategy for symptoms of CMDs (24). There are also extensive animal and human data pointing to the biological underpinnings of CMDs that are modulated by diet, with gut microbiota and inflammatory pathways gaining particularly attention (27, 28), in addition to brain plasticity pathways in humans (29). While the link between individual nutrients, supplements, and mental disorder treatment has been studied, this discussion aims to focus on prevention and treatment through a whole-of-diet approach to mental health (30–32). Results from ongoing and future intervention studies are essential to continuing to strengthen this evidence base, as well as advancing the diet–depression paradigm, and must be a funding and research priority (5).

## Why Act Now?

Given the strength and consistency of the epidemiological and animal evidence, coupled with the emerging evidence from intervention studies, we contend that diet should be considered a risk factor for the onset of CMDs, with public health messages and strategies developed that build on this new understanding. These will have the added benefit of targeting the non-communicable conditions that are so commonly comorbid with CMDs and which are responsible for a significant proportion of premature deaths worldwide (33). Moreover, dietary recommendations that focus on mental health may have more salience for the public, given that the possible consequences of unhealthy diets – heart disease, diabetes, and cancer – may be perceived as distal, while mental health is a far more proximal consequence for many. This is particularly the case for young people who are especially affected by both detrimental dietary changes (34) and mental health issues (35). Although obesity may be considered a key indicator of lifestyle, most epidemiological studies in this field demonstrated that the relationship between diet and depression exists independently of body mass index (11, 20, 36). Furthermore, improvements in mood may precede weight loss and may also provide more tangible and immediate benefits that encourage sustaining health behaviors, with weight or obesity management a possible downstream benefit (37–39).

Despite advances in aspects of mental health care, researchers and clinicians have highlighted the shortfalls of pharmacological or individualized clinical to patient care for CMDs (40). While intervention studies on the diet–depression paradigm remain a priority, the current evidence base meets key Bradford Hill Criteria, with few perceivable risks to impede action (41). Given the high prevalence and burden of CMDs, even slight improvements in depression through dietary intervention or prevention strategies may translate to large gains at the population level given that diet is a variable with 100% exposure.

## Implementing Dietary Improvement as a Public Health Strategy for Mental Health

There is much to be learned from previous public health movements that serve to guide the effective implementation of dietary improvement as a mental health strategy. While there is no country that has successfully managed to reverse its rising obesity trends (42), other public health campaigns that have been successful in improving health outcomes at a population level – such as folic acid supplementation during pregnancy or smoking cessation – have made changes using a top-down approach, supported by political changes to policies, practices, and taxation (43, 44). Implementing prevention strategies for mental health, and specific recommendations for how to proceed at community, academic, and government levels has been discussed in more detail elsewhere (5, 45, 46). Previous behavior change models have highlighted important periods for intervention, where targeted strategies may be more successful (47, 48). Previous research has demonstrated that *in utero*, early life, and adolescence are particularly important periods in determining future health and, thus, may be valuable target for public health campaigns. Given that pregnancy provides a unique “teachable moment,” where mothers are particularly open to receiving health advice and making behavior change, such a targeted approach to prevention of the CMDs may prove to be feasible (49) and have important implications for the mental health of offspring.

Poor quality diet should be included within the risk assessment for major depressive disorder (MDD) within clinical care settings, with dietary recommendations and dietetic services as central components of primary care for individuals diagnosed with depressive symptoms and MDD. Moreover, given the robust evidence base for physical activity as a protective and treatment factor in depression (50, 51), and the emerging evidence for the mental health benefits of smoking cessation (52), exercise recommendations and smoking cessation services should also be standard components of clinical care for at-risk patients and those with established CMDs. In other words, treatment for CMD should regard physical health as of equal importance as a treatment target when considering the mental health of a patient.

## Barriers to Implementation

There are several key considerations for implementing public health dietary interventions for mental health. One imperative is to demonstrate clear, objective benefits to motivate both policy, local and individual health behavior change. For this, a large-scale, worldwide, case-control study that calculates the percentage population attributable risk of poor diet to the incidence of CMDs will be required; akin to those that exist for heart disease, for example, INTERHEART study (2001). Additionally, well-enacted, robust interventions demonstrating efficacy and cost-effectiveness will be critical. At least one clinical trial is currently underway (53); however, more are needed. Importantly, public health interventions aimed at reducing NCD risk factors should also assess mental health outcomes and utilize findings to assess the effectiveness and cost-effectiveness of such interventions. As noted by many strategies in the past, single-approach or single-disciplinary approaches to complex, multi-modal problems have limited success, and there are demands for both systems-based interventions and monitoring systems and laws, regulations, and taxation changes to achieve traction in improving health at the population level (54).

While some studies have demonstrated the affordability of a healthy diet (55), financial incentives may be particularly important in groups with lower socioeconomic status (56). Finally, ensuring access to healthful foods for those in remote and highly disadvantaged areas is a critical challenge that should be prioritized, particularly given the burden of physical and mental health problems in such areas in addition to issues of food access.

## Conclusion

Despite increased awareness and attention, we have yet to effectively and consistency address the high prevalence and burden of CMDs. Improving diet quality has the important yet neglected potential for prevention and treatment of CMDs. Here, we contend that while intervention studies will be an essential component of this evidence base, the current understanding of the diet–CMD association calls for “consequentialist epidemiology,” with focus on the translation of diet-mental health research to action (57). Recognition of diet as a risk factor for CMDs and the development of public health strategies focused on dietary improvement are likely to positively address the global burden of both CMDs and NCDs. Effectively implementing dietary improvement as a public health strategy for mental health will require a multi-sectorial, multi-stakeholder approach (58), but offers substantial promise for improving outcomes for individuals and the wider community.

## Author Contributions

SD, AO, and FJ conceptualized the paper. SD wrote the first draft of the manuscript. AO and FJ made key contributions and revisions to the final version of the manuscript.

## Conflict of Interest Statement

The authors declare that the research was conducted in the absence of any commercial or financial relationships that could be construed as a potential conflict of interest.
